# Prediction of Chlorophyll Content in Multi-Temporal Winter Wheat Based on Multispectral and Machine Learning

**DOI:** 10.3389/fpls.2022.896408

**Published:** 2022-05-27

**Authors:** Wei Wang, Yukun Cheng, Yi Ren, Zhihui Zhang, Hongwei Geng

**Affiliations:** ^1^High-Quality Special Wheat Crop Engineering Technology Research Center, College of Agronomy, Xinjiang Agricultural University, Ũrũmqi, China; ^2^Department of Computer Science and Information Engineering, Anyang Institute of Technology, Anyang, China

**Keywords:** winter wheat, UAV, multispectral, machine learning, vegetation index

## Abstract

To obtain the canopy chlorophyll content of winter wheat in a rapid and non-destructive high-throughput manner, the study was conducted on winter wheat in Xinjiang Manas Experimental Base in 2021, and the multispectral images of two water treatments' normal irrigation (NI) and drought stress (DS) in three key fertility stages (heading, flowering, and filling) of winter wheat were obtained by DJI P4M unmanned aerial vehicle (UAV). The flag leaf chlorophyll content (CC) data of different genotypes in the field were obtained by SPAD-502 Plus chlorophyll meter. Firstly, the CC distribution of different genotypes was studied, then, 13 vegetation indices, combined with the Random Forest algorithm and correlation evaluation of CC, and 14 vegetation indices were used for vegetation index preference. Finally, preferential vegetation indices and nine machine learning algorithms, Ridge regression with cross-validation (RidgeCV), Ridge, Adaboost Regression, Bagging_Regressor, K_Neighbor, Gradient_Boosting_Regressor, Random Forest, Support Vector Machine (SVM), and Least absolute shrinkage and selection operator (Lasso), were preferentially selected to construct the CC estimation models under two water treatments at three different fertility stages, which were evaluated by correlation coefficient (*r*), root means square error (RMSE) and the normalized root mean square error (NRMSE) to select the optimal estimation model. The results showed that the CC values under normal irrigation were higher than those underwater limitation treatment at different fertility stages; several vegetation indices and CC values showed a highly significant correlation, with the highest correlation reaching.51; in the prediction model construction of CC values, different models under normal irrigation and water limitation treatment had high estimation accuracy, among which the model with the highest prediction accuracy under normal irrigation was at the heading stage. The highest precision of the model prediction under normal irrigation was in the RidgeCV model (*r* = 0.63, RMSE = 3.28, NRMSE = 16.2%) and the highest precision of the model prediction under water limitation treatment was in the SVM model (*r* = 0.63, RMSE = 3.47, NRMSE = 19.2%).

## Introduction

Soil plant analysis development (SPAD) can directly reflect the relative chlorophyll content in leaves (Netto et al., 2005), which was the most important pigment in photosynthesis, and its content was intimately related to the photosynthesis of plants (Zhang et al., 2019a) and the changes in its concentration directly affected the health of crops (Gitelson, 2005; Shestakova et al., 2020). Winter wheat was one of the world's major food crops. High yield and quality were the goals pursued by many breeders (Lesk et al., 2016; Sun et al., 2019). In recent years, extreme weather occurs frequently in the world and drought had directly affected wheat yields. It was estimated that drought and hot weather worldwide would reduce annual yields by 9–10% (Mondal et al., 2013). Crop growth can be predicted by constructing characteristics of nutrients and canopy spectra. Chlorophyll content played a large role in guiding the drought resistance and yield of wheat. Therefore, the study of chlorophyll content in wheat provided a basis for judging the growth of crops. Currently, remote sensing technology provided new ideas for the estimation of the chlorophyll content of crops, and the research mainly focused on spectral information indices and spectral information obtained by different sensors combined with data from the ground to predict the chlorophyll content.

Remote-sensing technology was currently showing strong competitiveness for precision agriculture in different experimental environments, especially that the convenient application of multispectral imaging technology on UAVs has accelerated the development of the technology (Kaivosoja et al., 2013; Yang et al., 2019). The acquisition of characteristic data on chlorophyll content at the ground level was usually destructive (Telmo, 2017). In addition, the ground acquired data was selected from a few limited points, and it was difficult to use these points to represent the characteristics of the whole area, so the acquisition of traditional ground phenology data was limited in scope. Remote-sensing data can be acquired at high throughput and large scale, but the influence of spatial image resolution made it difficult to grasp some local features, so UAV-based remote-sensing technology made up for this deficiency.

In recent years, with the compactness and convenience of UAVs and the ability to customize their missions, they had played an extremely important role in information-based agriculture (Sampson et al., 2003; Sun et al., 2021). The UAV was used as a spaceflight vehicle to carry various sensors, such as hyperspectral sensors, multispectral sensors, RGB cameras, and thermal infrared sensors (Zhang et al., 2021). Especially, hyperspectral and multispectral sensors were more common for the prediction of nutrient elements in crops. Currently, hyperspectral characterization data had been used to some extent for agricultural traits, but the popularity of hyperspectral use was far from multispectral due to some economic and complex reasons (Taghvaeian et al., 2012). Multispectral sensors carrying different wavelengths (Blue, Green, Red, Red_edge, and Nir) had a wide range of applications in many areas of crops. For instance, Bendig et al. (2015). predicted the biomass of crops by drones and obtained better results. The LAI, planting density, and photosynthetic characteristics of canola, barley, and wheat were well predicted by the UAV. In addition, the multispectral images from the UAV were a good reference for determining the emergence rate and rising potential of spring wheat. Moreover, there are also multispectral images from UAVs that serve as a good reference for the determination of seedling emergence, as well as the rise of spring wheat. Recently, some scholars have judged the maturity of wheat, as well as sorghum under drought conditions by UAV-based multispectral indices (Guillen-Climent et al., 2012; Verger et al., 2014; Jin et al., 2017). Hunt et al. (2013) constructed the Green Normalized Difference Vegetation Index (GNDVI) from multispectral images obtained by UAV and inversed the leaf area index of wheat through the vegetation index.

Combining ground phenotype and UAV multispectral image data for chlorophyll content inversion of crops was an innovative application of UAV multispectral sensors. Machine learning was the science of how to use computers to simulate or implement human learning activities, and it was the most intelligent feature of artificial intelligence, which can be used to integrate the data that had been generated for learning, and then, go on to make scientific predictions for the unknown world. The regression models within machine learning had shown strong data prediction capabilities for both linear and nonlinear, structured and unstructured data. For example, the least-squares algorithm, Random Forest algorithm, Support Vector Machine algorithm, decision tree algorithm, and Naïve Bayesian algorithm have been used to varying degrees in agricultural remote sensing(Garg et al., 2016; Grinberg et al., 2020; Shafiee et al., 2021).

Machine learning can not only perform predictive analysis on traditional data but also embodied great advantages in handling noise and anomalies data (Witten et al., 2016). For chlorophyll content prediction, multispectral images from UAV remote-sensing combined with machine learning algorithms provided excellent thoughts. Currently, machine learning combined with different vegetation indices had shown powerful advantages in agricultural remote-sensing, but studies combining a large number of machine learning algorithm models with preferred vegetation indices had rarely been reported. Next, classic machine learning algorithms for regression were employed to analyze the test data to find the best algorithm. Therefore, this article combined high-throughput UAV remote sensing images with preferential vegetation index and CC data to predict different fertility stages of winter wheat under different water treatments to achieve an intelligent level of wheat detection. This article focused on the following issues:(1) how to prefer vegetation indices for inverse model construction; (2) the effect of chlorophyll content on water response under different irrigation conditions; and(3) the response of different machine learning algorithms for different water treatments at different fertility stages to the prediction model of chlorophyll content in winter wheat.

## Materials and Methods

### Study Area and Experimental Design

The winter wheat field experiment was located in Manas, Xinjiang, China(86°12'52.2“N, 44°18'15.77”E), which had a mid-temperate continental arid semi-arid climate with severely cold winters, hot summers, dryness and low rainfall, sufficient sunshine, high evaporation, and low precipitation. In this study, there were 2 irrigation water treatments: DI and DS. A total of186 plots were selected for each irrigation treatment, 62 wheat varieties were selected for the experiment, each plot contained 1 variety, randomized group design, 3 repeats, 1-row zone(each of size 1.5 m × 0.3 m), and water restriction treatment was not watering during the wheat heading, flowering, filling, and maturity stages. The field management was by the local conventional cultivation management mode, and the wheat field grows well. Fertilizer application, drip irrigation, insect control, and weed control were also the same as the local field management.

### UAV Platform and Flight Configuration

In this study, the UAV platform was DJI Phantom 4 (Shenzhen Dajiang Technology Co. Ltd., Shenzhen, China), which carried a multispectral camera to collect multispectral images of the winter wheat canopy. The multispectral camera carried a camera with one RGB and multispectral channels with five wavelengths, centered at 450 nm (Blue), 560 nm (Green), 650 nm (Red), 730 nm (Red_edge), and 840 nm (Nir). Besides, the UAV is equipped with a light intensity sensor and a gray plate for radiation correction.

In the process of multispectral image acquisition, clear and windless weather at noon was selected, the UAV flew autonomously and recorded images according to the set route, and the multispectral camera lens was vertically downward, and the flight parameters were shown in Table 1.

**Table 1:** Unmanned aerial vehicle (UAV) flight parameters.

**Parameters**	**Parameter values**
Flight altitude	12 m
Flight Speed	5.4 km/h
Course overlap ratio	75%
Lateral overlap rate	75%
Spectral type	Blue, Green, Red, Red_edge, Nir

### Data Collection Plan

The data collection included CC of winter wheat canopy and UAV multispectral images. These data were collected at three different stages of wheat fertility: heading, flowering, and filling. Also, all the data in the acquisition plan contain two different water treatments. The specific acquisition plan for CC and UAV multispectral images were shown in Table 2.

**Table 2:** Manas UAV multispectral images and chlorophyll content (CC) data acquisition program.

**Date**	**Stages**	**Data**
2021.5.8	Heading	CC and Multispectral Images
2021.5.21	Flowering	CC and Multispectral Images
2021.5.28	Filling	CC and Multispectral Images

### Measurement of Chlorophyll Content

The correlation coefficients between SPAD values and chlorophyll content of wheat leaves had a significant level and can reflect the high and low levels of chlorophyll content of the crop (Netto et al., 2005). The measurement periods were three different fertility stages of wheat: heading stage, flowering stage, and filling stage. The relative chlorophyll content of different genotypes was measured simultaneously on the same day of the UAV flight by taking five uniformly growing wheat plants from each variety and using the SPAD-502 Plus chlorophyll meter, an instrument manufactured by Minolta Camera, Japan, which had been used by many scholars to obtain wheat CC data (Wang et al., 2012; Zhang et al., 2019b). The CC values were measured and recorded at the top, middle, and bottom of the flag leaf of selected winter wheat plants in the experiment field, and the average of the chlorophyll content of the three parts was taken as the CC values of the plant winter wheat, and then, the average of the CC values of five winter wheat plants was calculated as the CC values of this variety of winter wheat.

### Image Processing

**Figure 1:**
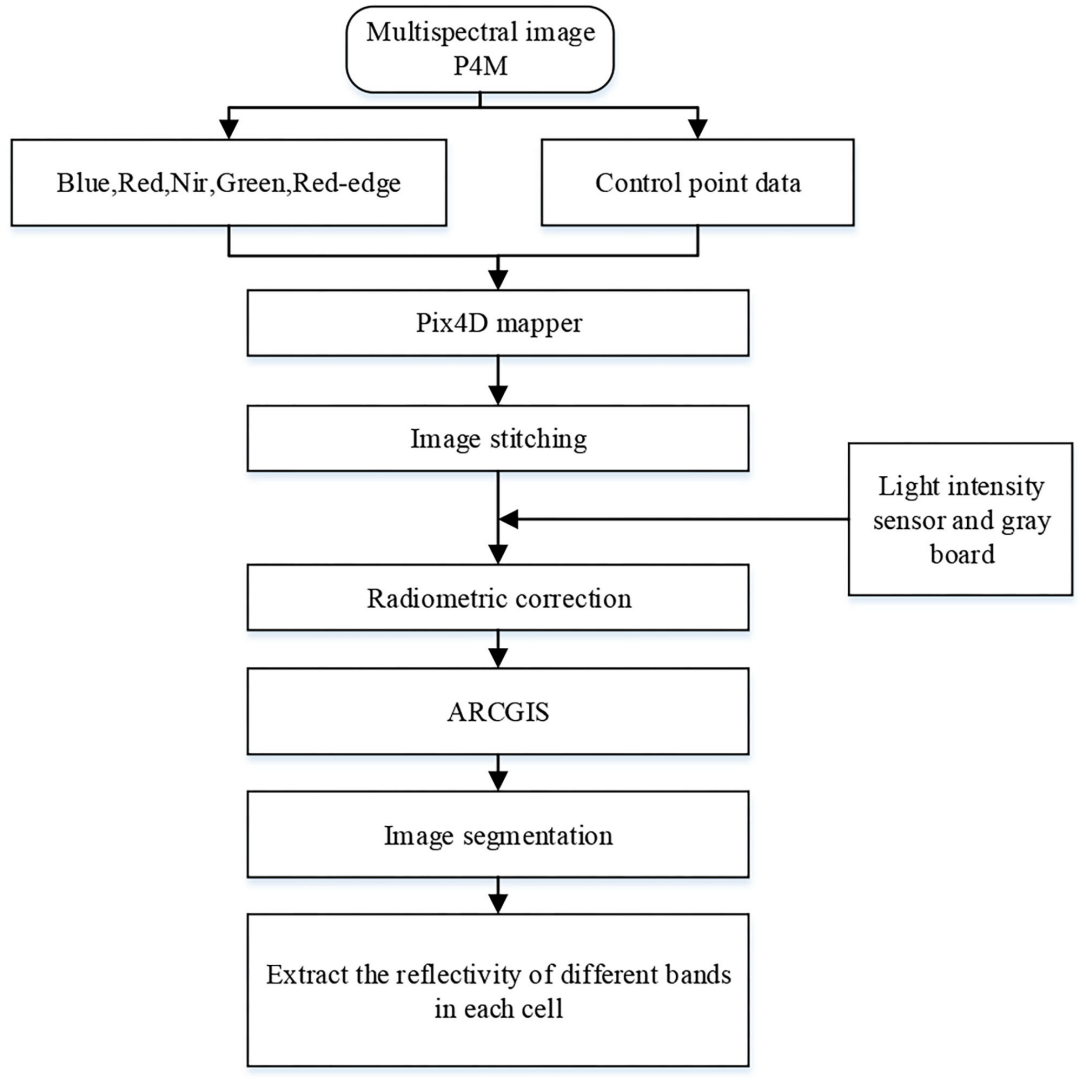
Flowchart of unmanned aerial vehicle (UAV) image processing.

In this study, Pix4Dmapper software (Version 1.4, Pix4d, Lausanne, Switzerland) (https://pix4d.com/) was used to stitch the acquired multispectral images of the UAV in 5 bands. The multispectral images were firstly corrected with the corresponding ground control point data to generate Digital Orthophoto Map (DOM); then, the reflectance correction of the multispectral images was performed with the gray plate to obtain the test site reflectance images, which were stored in TIF format; finally, ARCGIS software (Version 10.3.1, Esri, USA) (http://www.esri.com/arcgis/about-arcgis) was used to extract the vector surface of the cell and obtain the spectral reflectance images in 5 bands, and the average reflectance of this study area was extracted as the spectral reflectance of the sample in this band. The specific image processing flow was shown in Figure 1.

### Selection of Vegetation Index (VI)

The combination of changes in reflectance of different bands constitutes vegetation indices, which can reduce the degree of influence of factors, such as background soil on vegetation spectra, to a certain extent and improve the accuracy of estimating chlorophyll content. In this article, the importance of vegetation indices was evaluated by 13 vegetation indices in Table 3 except Green Band Optimized Soil Conditioning Vegetation Index (GOSAVI) using the Random Forest algorithm (Breiman, 2001). First, finding the top five vegetation indices in terms of contribution under different treatments, then, the vegetation indices were preferentially selected by combining the correlation between 14 vegetation indices and CC values, and, finally, the model inversion and prediction of CC values are carried out by using the preferential vegetation indices. In total, we selected 14 vegetation indices. The vegetation index calculation formula is shown in Table 3.

**Table 3:** Vegetation index and its calculation formula.

**Vegetation index**	**Formula to calculate**	**Reference**
Normalized vegetation index (NDVI)	*NDVI* = (*R*_*Nir*_−*R*_Re*d*_)/(*R*_*Nir*_+*R*_Re*d*_)	Schnell, 1974
Green Normalized Vegetation Index (GNDVI)	*GNDVI* = (*R*_*Nir*_−*R*_*Green*_)/(*R*_*Nir*_+*R*_*Green*_)	Wagner, 1996
Normalized Green and Blue Difference Index (NGBDI)	*NGBDI* = (*R*_*Green*_−*R*_*Blue*_)/(*R*_*Green*_+*R*_*Blue*_)	Hunt et al., 2005
Green Band Optimized Soil Conditioning Vegetation Index (GOSAVI)	*GOSAVI* = 1.16*[(*R*_*Nir*_−*R*_*Green*_)/(*R*_*Nir*_+*R*_*Green*_+0.16)]	Gilabert et al., 2002
Red edge optimized soil Regulating Vegetation Index (REOSAVI)	*REOSAVI* = 1.16*[(*R*_*Nir*_−*R*_*Red*_)/(*R*_*Nir*_+*R*_*Red*_+0.16)]	Kim et al., 1994
Optimization of Soil Conditioning Vegetation Index (OSAVI)	*OSAVI* = (*R*_*Nir*_−*R*_*Red*_)/(*R*_*Nir*_+*R*_*Red*_+0.16)	Rondeaux et al., 1996
Over Green Index (EXG)	*EXG* = 2*R*_*Green*_−*R*_*Red*_−*R*_*Blue*_	Torres-Sánchez et al., 2014
Green band atmospheric impedance vegetation index (VARIgreen)	*VARIgreen* = (*R*_*Green*_−*R*_*Red*_)/(*R*_*Green*_+*R*_*Red*_−*R*_*Blue*_)	Gitelson et al., 2002
Red-band atmospheric impedance vegetation index (VARIred)	*VARIred* = (*R*_*Red*_*edge*_−1.7*R_*Red*_+0.7*R_*Blue*_)/(*R*_*Red*_*edge*_+2.3*R_*Red*_−1.3*R_*Blue*_)	Gitelson et al., 2002
Modified simple ratio(MSR)	$MSR=\frac{R_{Nir}/R_{Red}-1}{\sqrt{R_{Nir}/R_{Red}}+1}$	Chen, 1996
Simple Ratio(SR)	*SR* = *R*_*Nir*_/*R*_*Red*_	Wagner, 1996
Green Chlorophyll Index(GCI)	*GCI* = *R*_*Nir*_/*R*_*Green*_−1	Gitelson, 2005
Normalized Difference Red-edge Index(NDREI)	*NDREI* = (_*R*_*Red*__*edge*_−*R*_*Green*_)/(_*R*_*Red*__*edge*_+*R*_*Green*_)	Muhammad et al., 2018
Normalized Green-Red Variance Index(NDRGI)	*NDRGI* = (*R*_*Green*_−*R*_*Red*_)/(*R*_*Green*_+*R*_*Red*_)	Schnell, 1974

*R_Blue_, R_Green_, R_Red_, R_Red_edge_ and R_Nir_ represent the reflectance of Blue wave band, Green band, Red band, Red_edge band, and Nir band, respectively*.

### Modeling Methods

**Figure 2:**
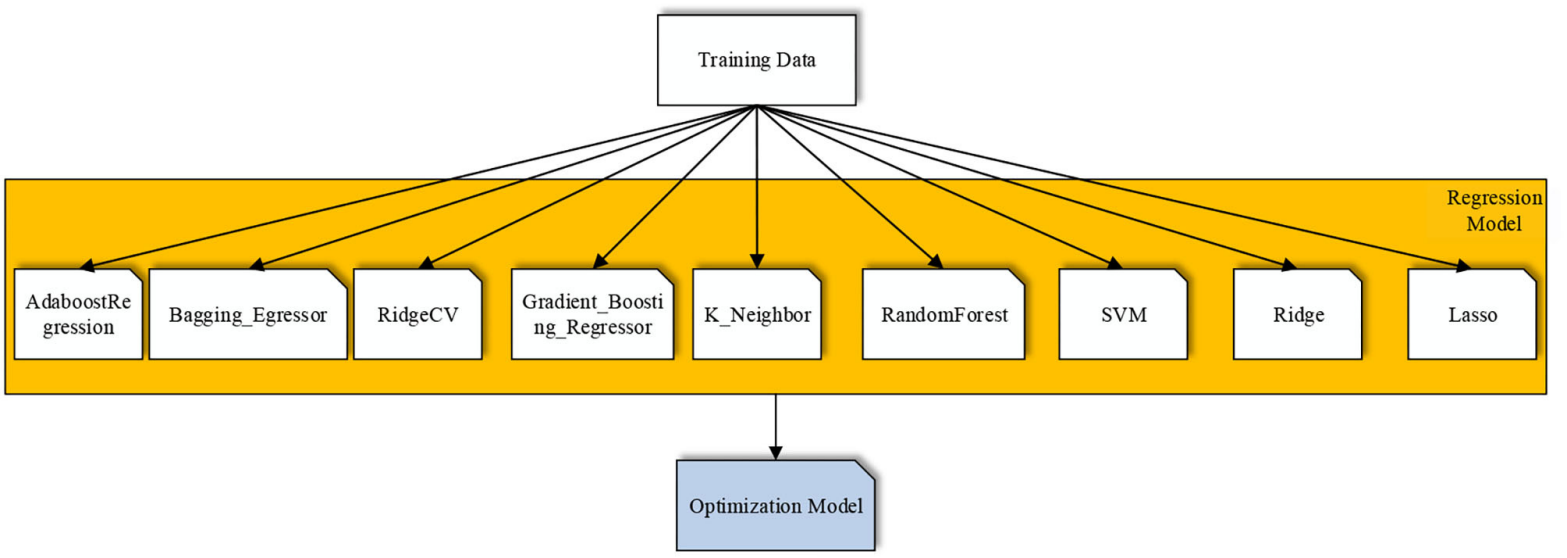
Flow chart of the inverse model of multiple machine learning algorithms.

Using remote-sensing images that predicted the chlorophyll content of ground crops by modeling, the analysis from mathematical models was the process of predicting chlorophyll by observing specific variables. Since the 1980s, machine learning has attracted wide interest in the artificial intelligence community as a way to achieve artificial intelligence, especially in the last decade or so, a rapid development of research work in the field of machine learning, and it has become one of the important topics of artificial intelligence. The algorithms of traditional machine learning had all shown strong advantages in data prediction regression. To predict the chlorophyll content of winter wheat, this study investigated the most classical machine learning algorithms of RidgeCV (Pelckmans et al., 2005), Ridge (Houwelingen, 1992), Adaboost Regression (Freund and Schapire, 1997), Bagging_Regressor (Hall and Turlach, 2007), K_Neighbor (Cover and Hart, 1967), Gradient_Boosting_Regressor (Friedman, 2001), Random Forest (Breiman, 2001), and SVM, Lasso (Tibshirani, 2011), and then, model inversion was performed with the studied data, and through an experimental cross-reference of the data, an attempt was made to find out the optimal regression learning algorithm that was most suitable for this study and provided model support for subsequent data prediction. The specific flow chart of the program implementation was shown in Figure 2.

To discuss the prediction model of CC in winter wheat at different fertility stages with different water treatments, data sets of different genotypes were used to construct the crop chlorophyll inversion model. In the process of model construction, the datasets were partitioned separately, and the datasets were distributed in a 7:3 ratio according to the random selection method of validation set and testers.

### Accuracy Evaluation

Pearson correlation coefficient (*r*), root mean square error (RMSE), and the normalized root mean square error (NRMSE) were used as evaluation indexes for different models, where the closer *r* was to 1, the lower the RMSE value indicates that the predicted and measured values of the model agreed. Also, the smaller the NRMSE value, the higher the accuracy of its estimation model and the better the effect. When the NRMSE is less than 10%, the model accuracy is very high, and the accuracy of the model is relatively high when the NRMSE is between 10 and 20%. The accuracy is at a normal level, when the NRMSE is between 20 and 30%, and when the NRMSE is more than 30%, the accuracy is poor. All data statistics experiments were implemented in Spyder by using Python 3.8.8 on a workstation with an Intel i7-6800 K 3.40 GHz CPU, 16 GB memory, and an Nvidia GeForce GTX 2080Ti graphics, running the Win10 operating system. Pandas 1.3.2, Matplotlib 3.4.2, and Scikit-Learn 0.24.2 were chosen for statistical analysis, correlation analysis, and significance of differences test for wheat CC.

## Results

### Reliability Verification of UAV Multispectral Imaging Data

**Figure 3:**
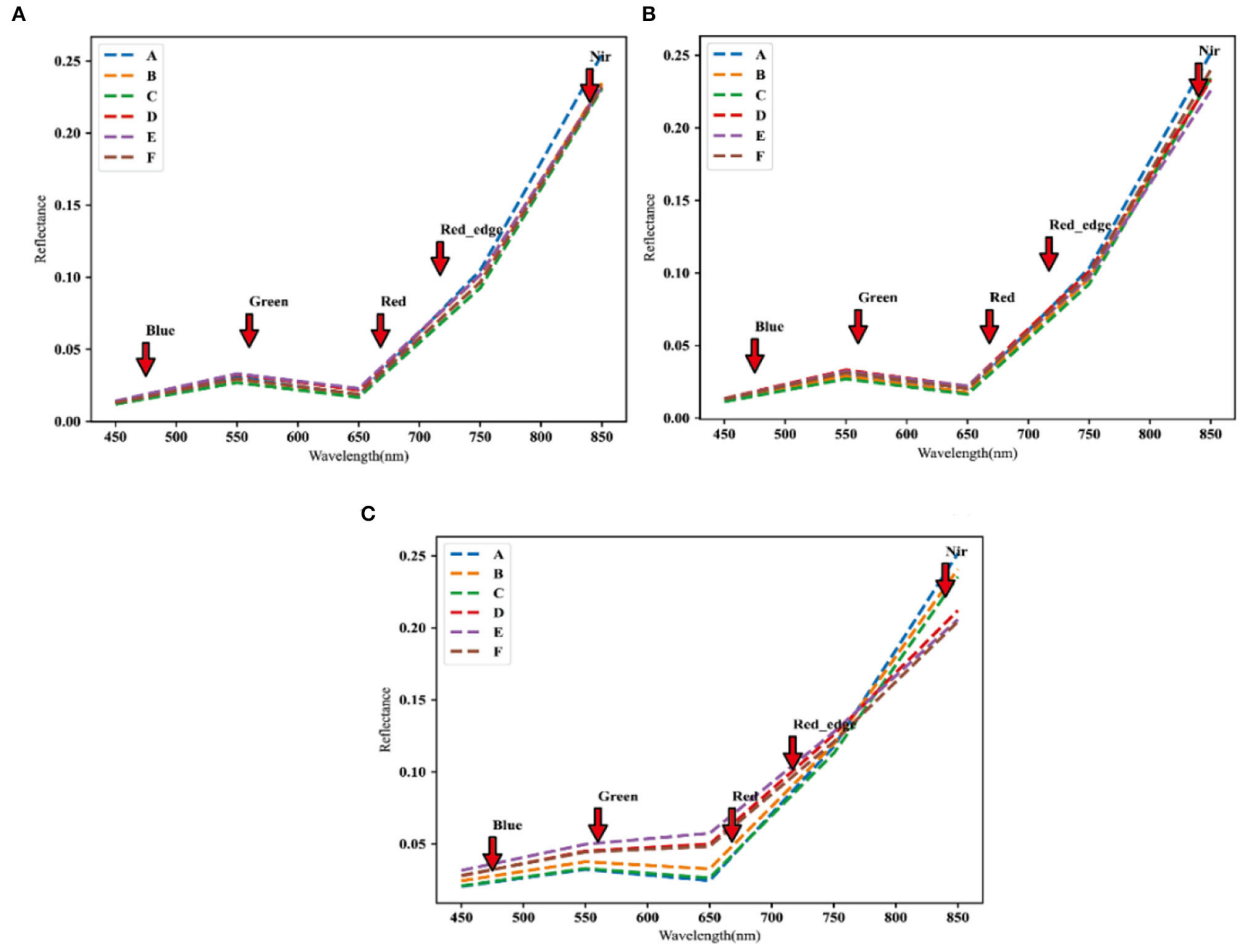
Different spectral reflectance maps of five bands of Manas winter wheat drones at different growing stages: **(A)** Manas winter wheat heading stage; **(B)** Manas winter wheat flowering stage; and **(C)** Manas winter wheat filling stage. A–C denote three replicates under normal irrigation; D–F denote three replicates under water-limited treatment.

The images of the UAV were extracted for the reflectance of different brands of spectra according to the plots, as shown in Figure 3, the spectral reflectance of different fertility periods were all in the range of 450–550-nm band, and the spectral reflectance curves of different regions showed an increasing trend, and the phenomenon of green light wave peak appeared around 550 nm, and this result was more consistent with the results of literature (Fu et al., 2019). The position of the green wave peak appeared differently in different fertility stages, and the wavelengths were from large to small in the filling stage, flowering stage, and heading stage. A red trough appeared between 630 and 670 nm, and the pattern of the red trough was the same as that of the green peak. In the range of 466–830 nm, the reflectance of multispectral data has high accuracy, and this result was more consistent with the results of literature (Aasen et al., 2015), and the five multispectral bands selected in this article were all in this range, which can estimate the canopy chlorophyll of winter wheat.

### Distribution of CC in the Winter Wheat

**Figure 4:**
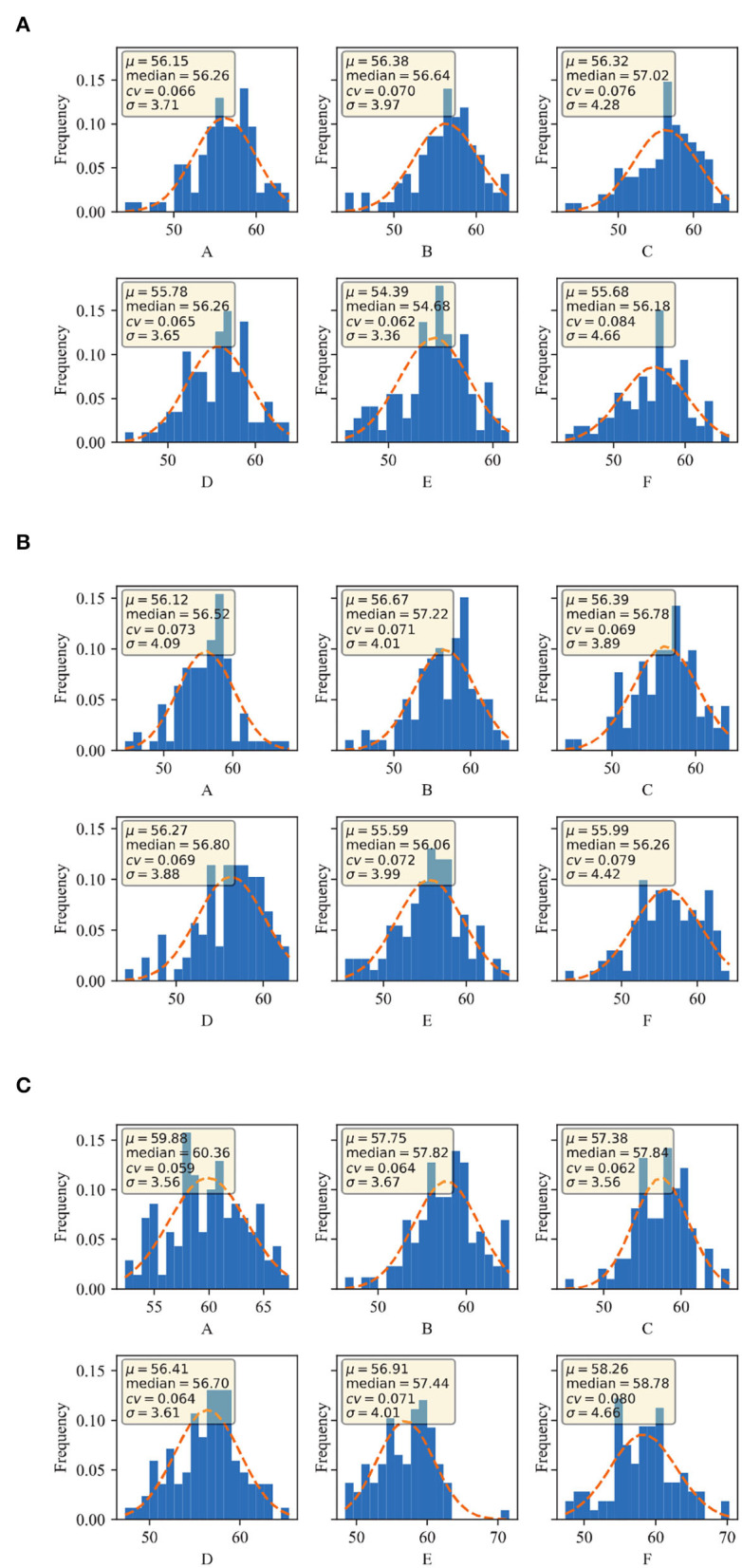
CC distribution of different growing stages of Manas winter wheat: **(A)** heading stage of Manas winter wheat; **(B)** flowering stage of Manas winter wheat; and **(C)** filling stage of Manas winter wheat. A–C denote three replicates under normal irrigation; D–F denote three replicates under water-limited treatment.

The CC values of winter wheat, under different water treatments of normal irrigation and water-limited treatment, were obtained at the heading, flowering, and filling stages, respectively. It was evaluated by four dimensions: mean value was expressed by μ, median by a median, coefficient of variation by cv, and standard deviation by σ. From Figure 4A, it can be seen that the mean values of CC in winter wheat at the heading stage were distributed between 54.39-56.38, the median ranged from 56.26-57.02, the σ ranged from 3.71-4.66, and the cv ranged 6.2%-8.4%. The cv of the CC under normal irrigation ranged from 6.6 to 7.6% as seen in Graph A, B, and C in Figure 4A. While the cv of the population under water limitation treatment ranged from 6.2 to 8.4%, as seen in Graphs D, E, and F. From Figure 4B, it can be shown that the mean values of CC in winter wheat at the flowering stage ranged from 55.59 to 56.39, the median ranged from 56.06 to 57.22, the σ ranged from 3.88 to 4. 22, and the cv ranged from 6.9 to 7.9%. The coefficient of variation of the population under normal irrigation ranged from 6.9%-7.3% as seen in plots A, B, and C in Figure 4B. While in plots D, E, and F, the cv of the population under water limitation treatment ranged from 6.9 to 7.9%. From Figure 4C, it can be observed that the mean values of CC in winter wheat at the filling stage ranged from 56.41 to 59.88, the median ranged from 56.7 to 60.36, the σ ranged from 3.56 to 4.66, and the cv ranged from 5.9 to 8%. The cv of the population under normal irrigation ranged from 5.9 to 6.2% as seen in plots A, B, and C in Figure 4C, while in plots D, E, and F, the cv of the population under water limitation treatment ranged from 6.4 to 8%.

In summary, the overall dispersion and variation of the data were large, indicating that the population showed great variation in CC at the heading, flowering, and filling stages, and indicating that the population was rich in genetic variation. In terms of water and drought treatments, the range of variation was 1.2% for the water treatment over normal irrigation at the heading stage, 2.6% for the water treatment over normal irrigation at the flowering stage, and 1.3% for the water treatment over normal irrigation at filling stage. The range of variation became larger from the heading stage to the flowering stage, and then gradually decreased with the extension of the reproductive period.

### Preferred Vegetation Index (VI)

**Figure 5:**
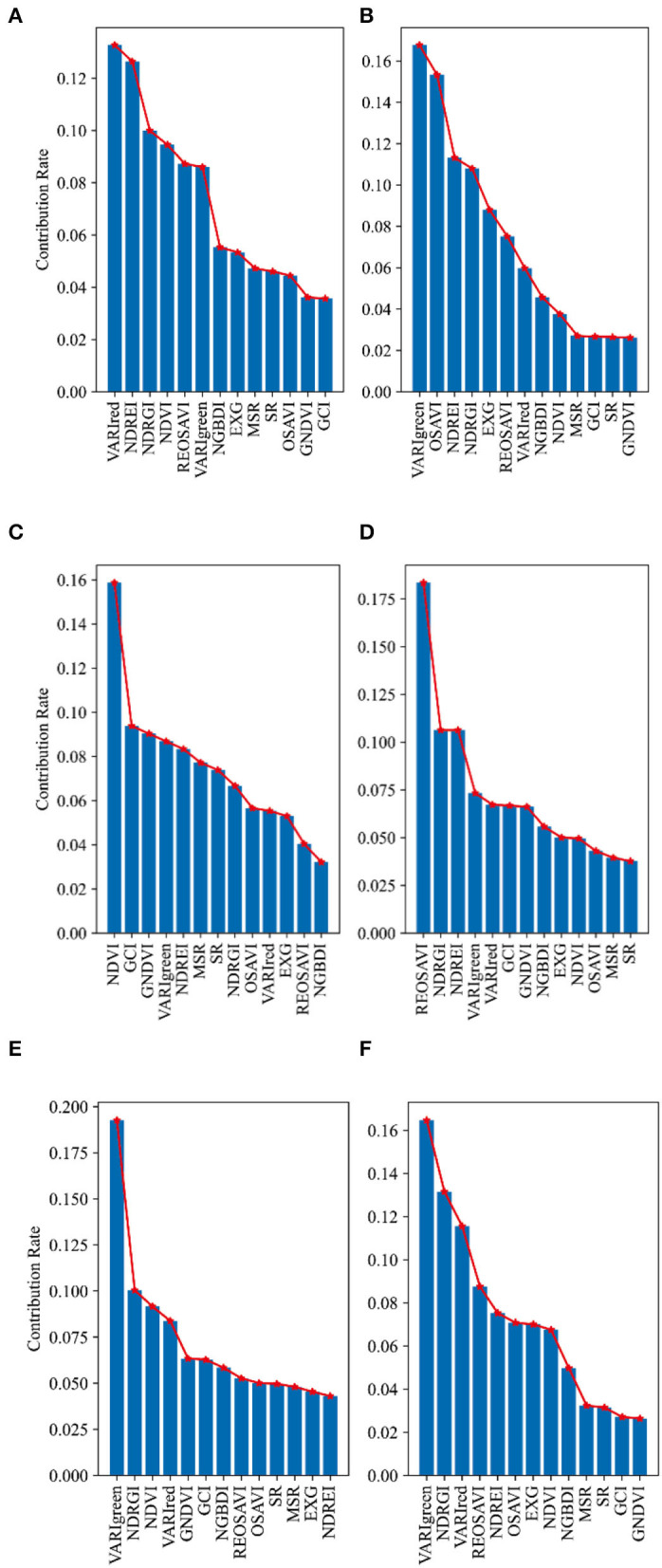
Contribution rate distribution of vegetation indices relative to CC at different growing stages of winter wheat: **(A)** normal irrigation at heading stage of Manas winter wheat; **(B)** water limited treatment at heading stage of Manas winter wheat; **(C)** normal irrigation at flowering stage of Manas winter wheat; **(D)** water limited treatment at flowering stage of Manas winter wheat; **(E)** normal irrigation at filling stage of Manas winter wheat; and **(F)** water limited treatment at filling stage of Manas winter wheat. All vegetation indices that appear in the figure are explained in Table 3.

Many selections of vegetation indices were made based on empirical values, and the visualization of the selection process was rarely given. In this study, experiments on the contribution of vegetation indices relative to CC were conducted in combination with the random forest algorithm at the heading, flowering, and filling stages of wheat under normal irrigation and water-limited treatment, respectively. It can be seen from Figure 5 that the contribution of vegetation index to CC was different under different water treatments in different periods. From Figure 5A, it can be seen that the magnitude of contribution under normal irrigation at the heading stage was as follows: REOSAVI>VARIgreen>NDREI>NDRGI>NDVI>VARIred> NGBDI>OSAVI>MSR>SR>EXG>GCI>GNDVI; from Figure 5B, we can see that the magnitude of contribution under water irrigation at the heading stage was: VARIgreen> OSAVI> NDREI> NDRGI> EXG> REOSAVI> VARIred> NGBDI> NDVI> MSR> GCI> SR> GNDVI. As seen in Figure 5C, the magnitude of contribution under normal irrigation during flowering was in the following order: NDVI>GCI>VARIgreen>NDREI>MSR>SR>NDRGI> OSAVI>VARIred>EXG>REOSAVI>.

NGBDI; as seen in Figure 5D, the magnitude of contribution under water irrigation during flowering was in the following order: REOSAVI > NDRGI > NDREI > VARIgreen> VARIred > GCI> GNDVI> NGBDI> EXG> NDVI> OSAVI> MSR> SR. From Figure 5E, it can be observed that the magnitude of contribution under normal irrigation during the filling period is in the following order: VARIgreen > NDRGI > NDVI > VARIred > GNDVI > GCI > NGBDI > REOSAVI > OSAVI > SR > MSR > EXG > NDREI; from Figure 5F, it can be seen that the magnitude of contribution under water irrigation during the filling period is in the following order: VARIgreen > NDRGI > VARIred > REOSAVI > NDREI > OSAVI > EXG > GCI > GNDVI.

In general, the contribution of vegetation indices under two different water treatments can be found in winter wheat at the heading stage, and the overall contribution was ranked in the top 5 with three vegetation indices, VARIgreen, NDREI, and NDRGI, which were used as the priority vegetation indices when the model was constructed at the heading stage. In the flowering stage of winter wheat, the contribution of vegetation indices under two different water treatments was found to be different, and the overall contribution was ranked in the top 5 with two vegetation indices, VARIgreen and NDRGI, which were used as the priority vegetation indices in the model construction of the flowering stage. In winter wheat, the contribution rates of vegetation indices under two different water treatments were found to be different in the filling stage, and the overall contribution rates were ranked in the top 5 with three vegetation indices, VARIgreen, NDRGI, and VARIred, which were used as the priority vegetation indices in the model construction of the filling stage.

### Correlation Analysis Between SPAD and Vegetation Index of Winter Wheat

**Figure 6:**
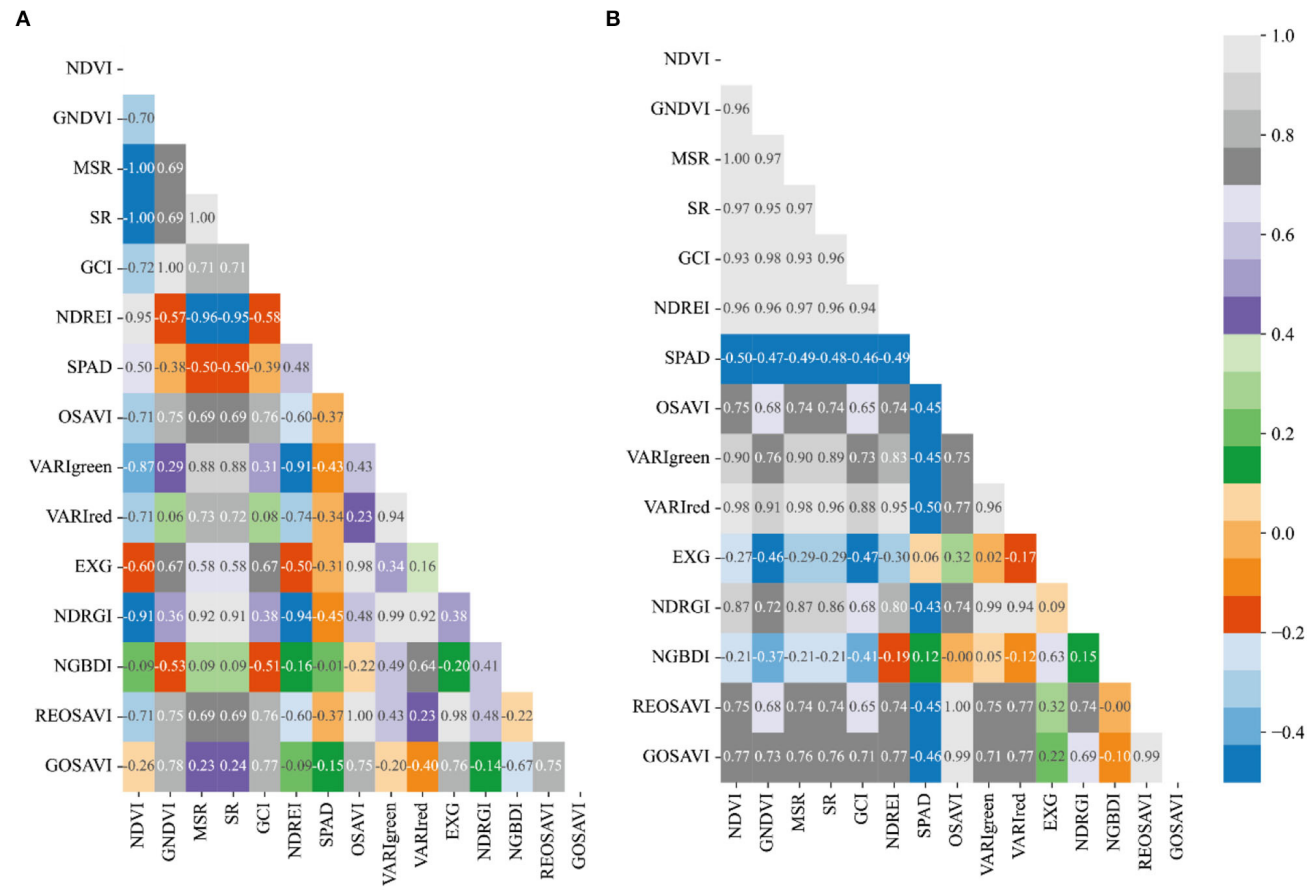
Plot of correlation between different vegetation indices and CC of winter wheat during heading stage: **(A)** normal irrigation of Manas winter wheat during heading stage, and **(B)** water limited treatment of Manas winter wheat during heading stage. All vegetation indices that appear in the figure are explained in Table 3.

**Figure 7:**
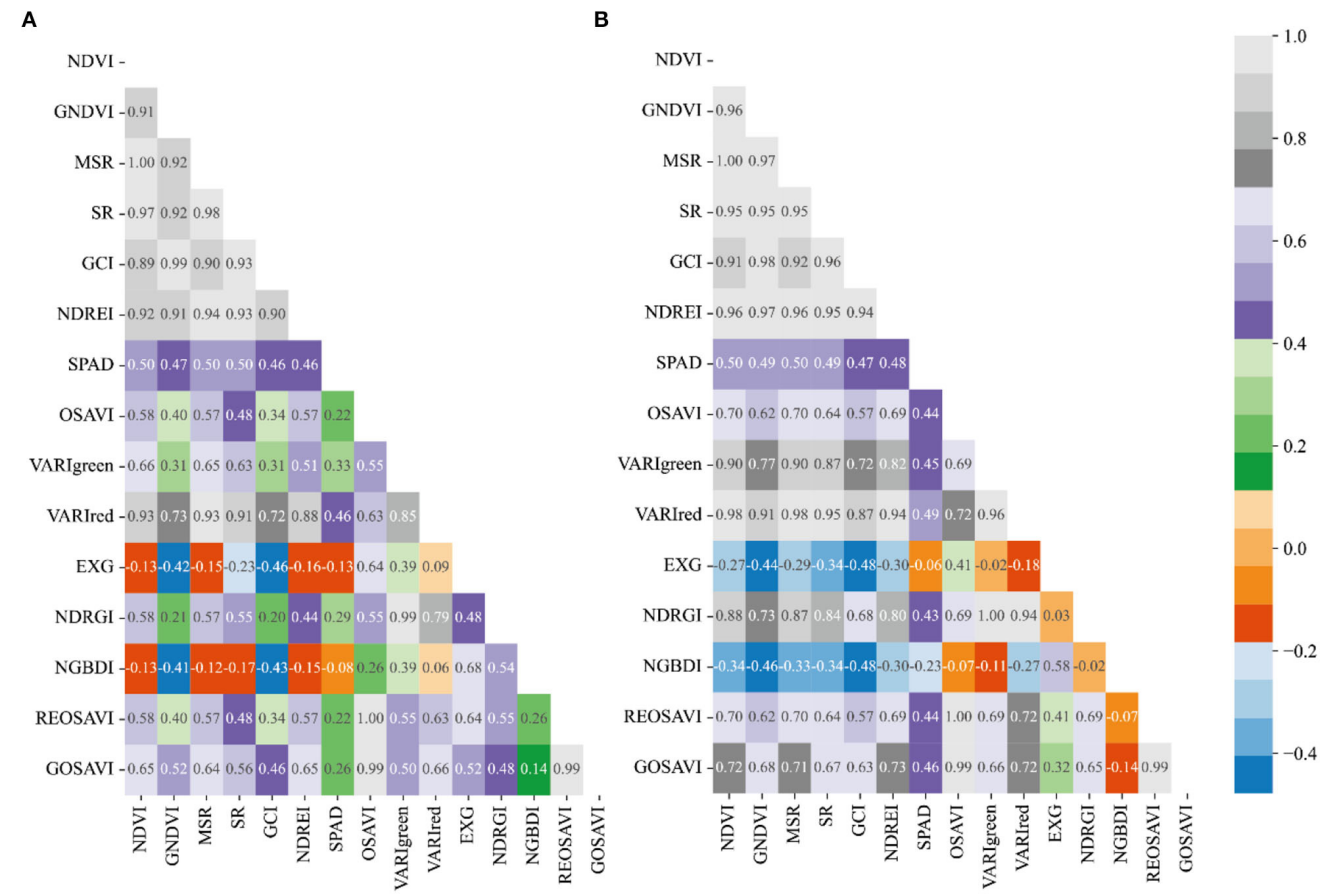
Plot of correlation between different vegetation indices and CC of winter wheat during flowering stage: **(A)** Normal treatment of Manas winter wheat during flowering stage, and **(B)** water limited treatment of Manas winter wheat during flowering stage. All vegetation indices that appear in the figure are explained in Table 3.

**Figure 8:**
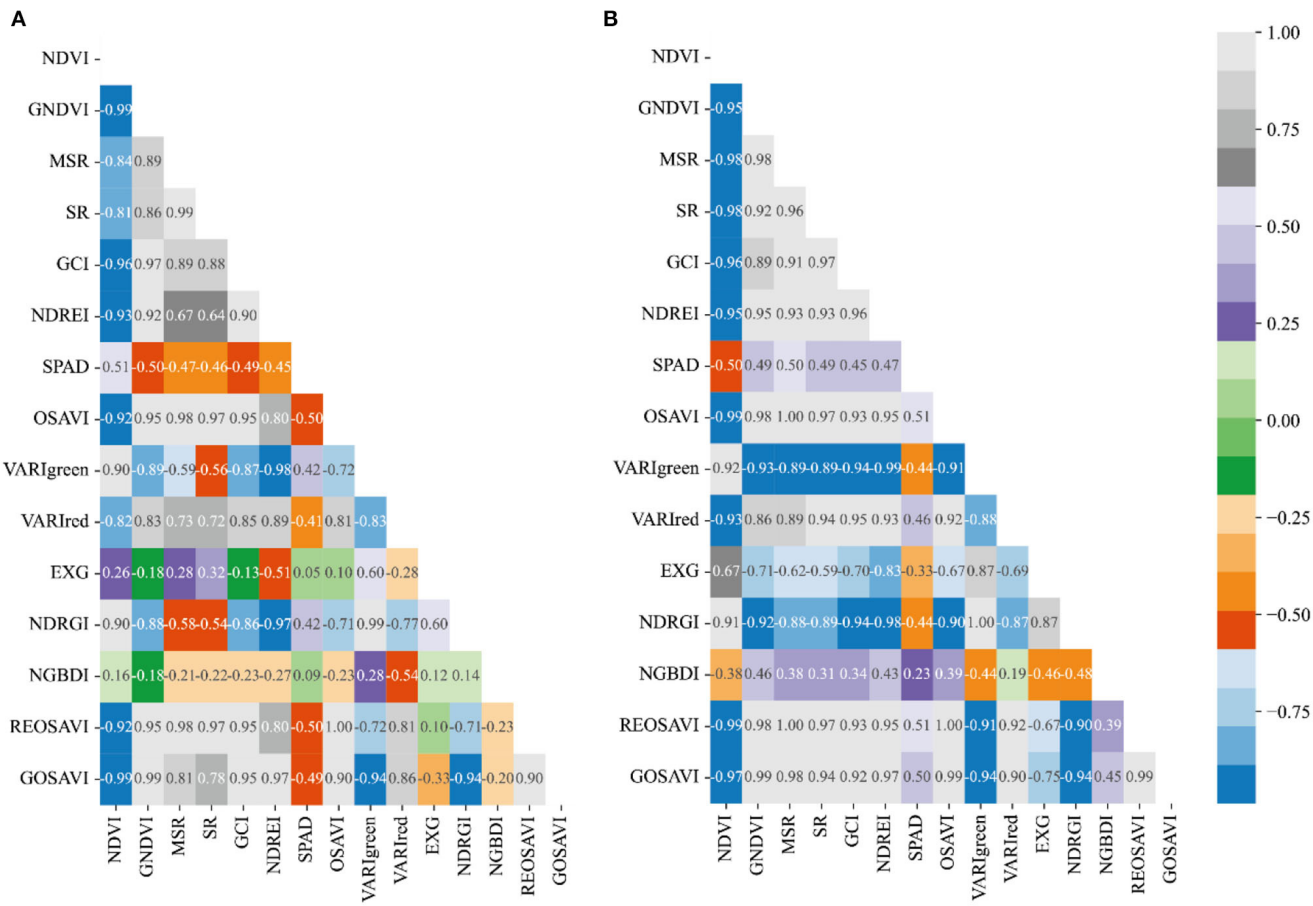
Plot of correlation between different vegetation indices and CC of winter wheat during the filling period: **(A)** Normal treatment of Manas winter wheat during the filling period, and **(B)** Water treatment of Manas winter wheat during the filling period. All vegetation indices that appear in the figure are explained in Table 3.

The spectral parameters of the three fertility stages of heading, flowering, and filling were correlated with winter wheat CC and the results were shown in Figures 6–8. From Figure 6A, it can be seen that most of the spectral vegetation indices selected under normal irrigation at the heading stage reached highly significant levels (*p* < 0.0001). Among them, Normalized Green and Blue Difference Index (NGBDI) showed no significant correlation during the heading period, while the rest of the parameters showed correlation, among which the highest positive correlations were NDVI, MSR, and SR with correlation coefficients *r* reaching.5, followed by NDREI with correlation coefficient *r* reaching.48. It can be seen from Figure 6B that most of the selected spectral vegetation indices under water limitation treatment reached highly significant levels (*p* < 0.0001). Among them, Over Green Index (EXG) and NGBDI showed no significant correlation at the heading stage, while the rest of the parameters showed correlation, with the highest negative correlation being NDVI and VARIred, with a correlation coefficient *r* reaching −0.5, followed by MSR and NDREI, with a correlation coefficient *r* reaching −0.49.

While the correlation coefficient *r* of VARIgreen in section Preferred Vegetation Index (VI), using the preferred vegetation index of the random forest, was −0.43 under normal irrigation and −0.45 under water-limited treatment; the correlation coefficient *r* of NDREI was 0.48 under normal irrigation and −0.49 under water-limited treatment; the correlation coefficient *r* of NDRGI was −0.45 under normal irrigation and −0.43 under water limitation treatment; all three vegetation indices reached a highly significant level (*p* < 0.0001).

Most of the spectral vegetation indices selected under normal irrigation during flowering reached a highly significant level (*p* < 0.0001) as can be seen in Figure 7A. Of these, EXG and NGBDI showed no significant correlation, while the rest of the parameters showed correlation, with the highest positive correlation being NDVI and MSR, with SR correlation cefficient *r* reaching 0.5, followed by VARIred, with correlation coefficient *r* reaching 0.46. From Figure 7B it can be seen that most of the spectral vegetation indices selected under water limitation treatment reached highly significant levels (*p* < 0.0001). Among them, EXG showed no significant correlation, while the rest of the parameters showed correlation, with the highest positive correlation being NDVI and MSR, with a correlation coefficient *r* reaching 0.5, followed by GNDVI, SR, and VARIred, with a correlation coefficient *r* reaching −0.49.

In contrast, in the preferred vegetation index using the random forest in Section Preferred Vegetation Index, the correlation coefficient *r* for VARIgreen was 0.45 and 0.33, respectively, under normal irrigation and water-limited treatment; the correlation coefficient *r* for Red Edge Index (NDREI) was 0.46 under normal irrigation and 0.48 under water-limited treatment, and both 2 vegetation indices reached a highly significant level (*p* < 0.0001).

It is evident from Figure 8A that most of the spectral vegetation indices selected under normal irrigation during the filling period reached a highly significant level (*p* < 0.0001). The positive correlation was highest for NDVI with a correlation coefficient *r* of 0.51, followed by VARIgreen with a correlation coefficient *r* of 0.42, and the negative correlation was highest for GNDVI with a correlation coefficient *r* of −0.50. It can be seen from Figure 8B that the spectral vegetation indices selected under water limitation treatments all reached highly significant levels (*p* < 0.0001). All the vegetation indices selected under the water limitation treatment reached a highly significant level (*p* < 0.0001). The highest positive correlations were OSAVI and REOSAVI with a correlation coefficient *r* of 0.51, followed by MSR and GOSAVI with a correlation coefficient *r* of 0.50. The highest negative correlation was NDVI, and the correlation coefficient *r* reached −0.50.

Meanwhile, the correlation coefficient *r* of VARIgreen under normal irrigation and −0.44 under water-limited treatment in the preferred vegetation index using the random forest in section Preferred Vegetation Index was 0.42; the correlation coefficient *r* of NDRGI was 0.42 under normal irrigation and −0.44 under water-limited treatment, and the correlation coefficient *r* of VARIred under normal irrigation was −0.41 under normal irrigation and.46 underwater limitation treatment, all three vegetation indices reached significant levels (*p* < 0.0001).

Therefore, the correlation analysis of vegetation indices and CC of winter wheat for the three fertility periods of winter wheat was combined, and the correlations of the vegetation indices preferred in the previous section all reached significant levels, and in addition, the model estimation was carried out by combining the vegetation indices with the highest correlation in that fertility period.

### Algorithm Development for CC Estimation

The model inversions were conducted using nine machine learning algorithms, Adaboost Regression, Bagging_Regressor, Gradient_Boosting_Regressor, K_Neighbor, Random Forest, SVM, Lasso, RidgeCV, and Ridge, for the SPAD values of winter wheat at the heading, flowering, and filling stages under two water treatments. The results in Table 4 show that the correlation coefficients between predicted and true values under normal irrigation at the heading stage ranged from 0.36 to 0.63 for *r*, 3.28–3.67 for RMSE, and 16.2–18.1% for NRMSE. The highest correlation was the RidgeCV model with correlation coefficient *r* = 0.63, which had RMSE = 3.28 and NRMSE = 16.2% for both Random Forest and RidgeCV in terms of model accuracy. Overall, it shows that the RidgeCV model has the best prediction accuracy and prediction effect under normal water treatment at the heading stage. In contrast, the correlation coefficients *r* between the predicted and true values under the water-limited treatment at the heading stage ranged from 0.41 to 0.63, RMSE from 3.44 to 3.95, and NRMSE from 18.8 to 21.9%. The highest prediction correlation is the SVM model with a correlation coefficient of *r* = 0.63, RMSE = 3.47 and NRMSE = 19.2%, which is still very good in terms of prediction accuracy. In terms of the prediction accuracy of the model, Adaboost Regression has the smallest NRMSE of 18.8%, while the prediction correlation is *r* = 0.60.

**Table 4:** Model analysis of CC for vegetation index prediction at the heading stage.

**Models**	**DI**			**DS**		
	***r***	**RMSE**	**NRMSE (%)**	***r***	**RMSE**	**NRMSE (%)**
Adaboost Regression	0.44	3.5	17.3	0.60	3.39	18.8
Bagging_Regressor	0.49	3.39	16.8	0.48	3.77	20.9
Gradient_Boosting_ Regressor	0.36	3.67	18.1	0.41	3.95	21.9
K_Neighbor	0.50	3.38	16.7	0.58	3.44	19.1
Random Forest	0.55	3.28	16.2	0.56	3.5	19.4
SVM	0.62	3.58	17.3	0.63	3.47	19.2
Lasso	0.61	3.37	16.7	0.60	3.48	19.3
RidgeCV	0.63	3.28	16.2	0.61	3.44	19.1
Ridge	0.61	3.47	17.1	0.60	3.47	19.2

*DI, normal irrigation; DS, limited water treatment*.

The results of the model analysis of the predicted CC of vegetation index under normal irrigation and water limitation treatments during flowering are shown in Table 5. The correlation coefficients *r* between predicted and true values under normal irrigation ranged from 0.27 to 0.50, RMSE from 2.79 to 3.20, and NRMSE from 17 to 19.5%. The highest model prediction correlation is the SVM model, which has a correlation coefficient of *r* = 0.50, RMSE = 2.79 and NRMSE = 17%, and the SVM also has the lowest NRMSE in terms of model accuracy. The correlation coefficients *R*^2^ between predicted and true values under water-limiting treatment ranged from 0.42 to 0.50, RMSE from 2.90 to 3.03, and NRMSE from 19.1 to 20.3%. The highest correlation predicted by the models was the Bagging_Regressor model, which had a correlation coefficient of *r* = 0.50, RMSE = 2.90, and NRMSE = 19.1%, with Bagging_Regressor having the lowest NRMSE as far as the accuracy of the model is concerned. Overall, it shows that the Bagging_Regressor model has the best prediction accuracy and prediction under flowering duration water treatment.

**Table 5:** Model analysis of CC for predicting vegetation index during flowering.

**Models**	**DI**			**DS**		
	***r***	**RMSE**	**NRMSE (%)**	***r***	**RMSE**	**NRMSE (%)**
Adaboost Regression	0.34	3.09	18.8	0.44	3.03	19.9
Bagging_Regressor	0.40	2.96	18.0	0.50	2.90	19.1
Gradient_Boosting_ Regressor	0.49	2.81	17.1	0.43	3.08	20.3
K_Neighbor	0.27	3.20	19.5	0.44	3.02	19.9
Random Forest	0.46	2.83	17.2	0.45	3.01	19.8
SVM	0.50	2.79	17.0	0.46	2.98	19.6
Lasso	0.47	2.81	17.1	0.43	3.02	19.8
RidgeCV	0.47	2.81	17.1	0.42	3.03	20.0
Ridge	0.47	2.81	17.1	0.44	3.00	19.7

*DI, normal irrigation; DS, limited water treatment*.

Analysis of the model for predicting CC of vegetation index under normal irrigation and water limitation treatments during the irrigation period is shown in Table 6. The correlation coefficients *r* between the predicted and true values under normal irrigation ranged from 0.21 to 0.43, RMSE from 2.91 to 3.49, and NRMSE from 21.6 to 25.8%. The model with the highest model prediction correlation is the SVM, which has a correlation coefficient of *r* = 0.43, RMSE = 2.91, and NRMSE = 21.6%, and the SVM also has the lowest NRMSE in terms of the accuracy of the model. The correlation coefficients *r* between predicted and true values under water-limiting treatment ranged from 0.32 to 0.51, RMSE from 3.57 to 4.17, and NRMSE from 16.3 to 19%. The highest correlation predicted by the models was the SVM model, which had a correlation coefficient of *r* = 0.51, RMSE = 3.57, and NRMSE = 16.3%, and the SVM had the lowest NRMSE in terms of the accuracy of the model. Overall it shows that the SVM model has the best prediction accuracy and prediction under normal irrigation and water limitation treatment during the irrigation period.

**Table 6:** Determination coefficient (*r*), root mean square error (RMSE), and relative error (RE) of the algorithms for modeling estimation of chlorophyll content (CC) of wheat in different filling period and normal irrigation (NI) and drought stress (DS) conditions.

**Models**	**DI**			**DS**		
	***r***	**RMSE**	**NRMSE (%)**	***r***	**RMSE**	**NRMSE (%)**
Adaboost Regression	0.34	3.08	22.8	0.35	4.08	18.6
Bagging_Regressor	0.21	3.49	25.8	0.37	4.04	18.4
Gradient_Boosting_ Regressor	0.27	3.36	24.9	0.32	4.17	19.0
K_Neighbor	0.41	2.97	22.0	0.43	3.78	17.2
Random Forest	0.26	3.28	24.3	0.40	3.91	17.8
SVM	0.43	2.91	21.6	0.51	3.57	16.3
Lasso	0.41	2.96	21.9	0.48	3.69	16.8
RidgeCV	0.41	2.94	21.8	0.48	3.69	16.8
Ridge	0.40	2.96	22.0	0.49	3.68	16.8

*DI, normal irrigation; DS, limited water treatment*.

In general, among the prediction models of CC using nine machine learning algorithms for three different fertility stages, namely, heading, flowering, and filling, the correlation coefficients of the RidgeCV model under normal irrigation and the SVM model underwater restriction treatment were the highest in the heading stage; the correlation coefficients of the SVM model under normal irrigation and the SVM model underwater restriction treatment were the highest in the flowering stage; the correlation coefficients of the Bagging_Regressor model were the highest in both normal irrigation and water restriction treatments. The correlation coefficient of the SVM model was the highest under normal irrigation and the SVM model was the highest underwater restriction. In terms of prediction accuracy, Random Forest and RidgeCV models had the highest prediction accuracy under normal irrigation in the heading stage, and the Adaboost Regression model had the highest prediction accuracy under water restriction treatment; the SVM model had the highest prediction accuracy under normal irrigation in the flowering stage, and Bagging_Regressor model had the highest prediction accuracy under water restriction treatment. The highest prediction accuracy was achieved by the SVM model under normal irrigation and water restriction treatment at the flowering stage, and the highest prediction accuracy was achieved by the SVM model underwater restriction treatment.

## Discussion

### Effect of Water and Drought Treatment on Chlorophyll

In drought environments, plants themselves evolve a series of mechanisms for self-protection and adaptation and resistance to unfavorable environmental stresses, and their phenotypic characteristics are significantly altered to minimize the impact of the adverse environment on their growth and development. At the same time, drought stress causes complex effects on the population structure and physiology of crops in various ways (Roessner, 2012). Concerning the reproductive stages of wheat, the whole reproductive period is divided into four stages: early-stage (sowing-pulling), developmental stage (pulling-heading), middle stage (pulling-potting), and late-stage (potting-harvest). Previous studies on the effects of drought stress on different fertility stages of wheat have suggested that drought stress affects the internal physiological and biochemical phenotypes of wheat to different degrees, and these changes are manifested in changes in chlorophyll content (Cao, 2010), indicating that chlorophyll content is closely related to drought resistance and yield traits in wheat.

**Figure 9:**
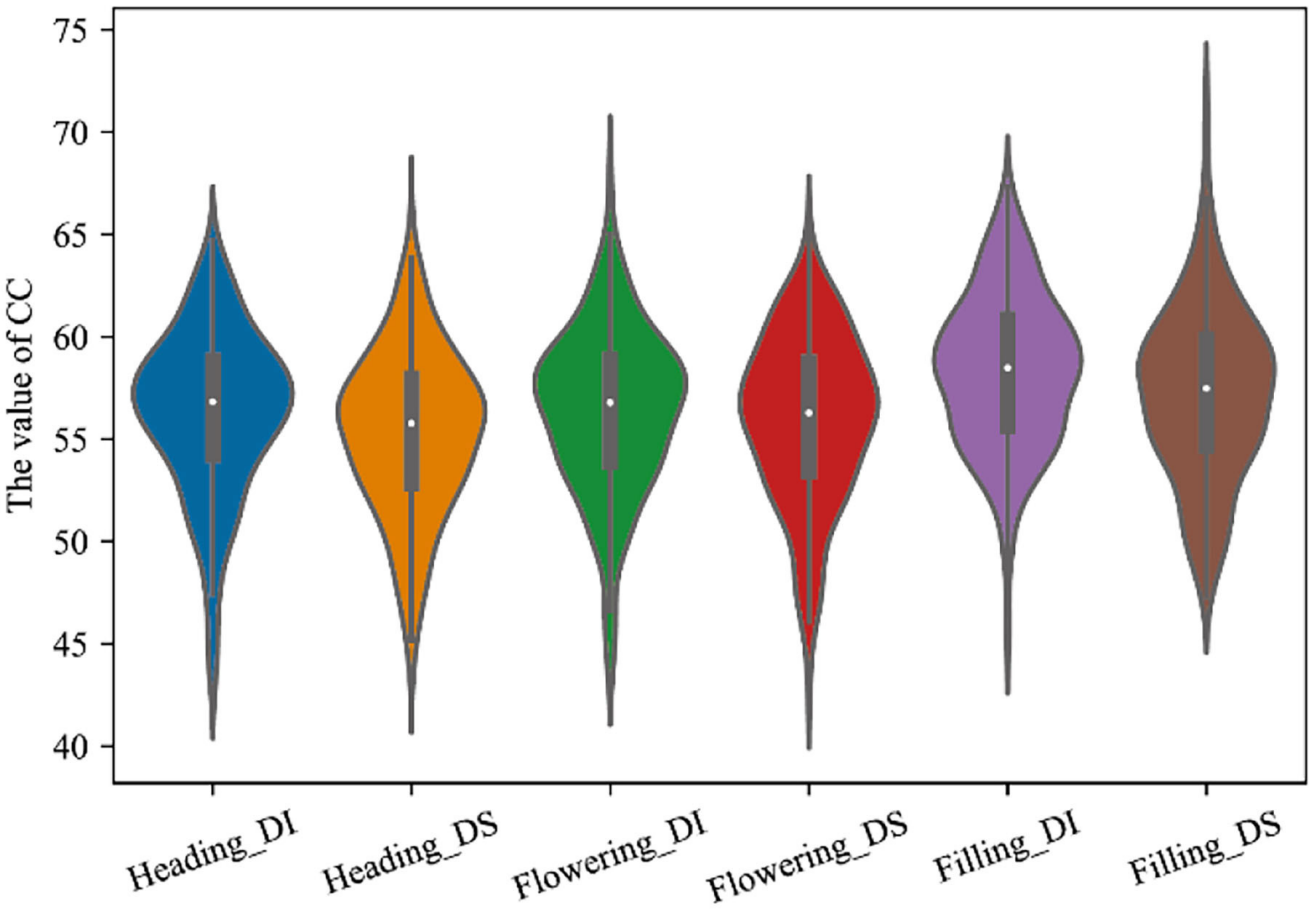
CC distribution of winter wheat at different fertility stages under two water treatments. Heading_DI, Heading_DS, Flowering_DI, Flowering_DS, Filling_DI, and Filling_DS refer to normal treatment at heading stage, water treatment at heading stage, normal treatment at flowering stage, water treatment at flowering stage, normal treatment at filling stage, and water treatment at filling stage, respectively.

In this study, for the analysis of canopy chlorophyll content of winter wheat, the results in Figure 9 showed that normal irrigation conditions increased the canopy chlorophyll content of wheat from the heading stage to the filling stage very significantly, compared with drought stress. In addition, chlorophyll is the most important pigment for photosynthesis, which affects the physiological and biochemical processes in the crop under drought stress. Drought stress causes reactive oxygen species produced by the plant body to disrupt cell membranes, which hinders chlorophyll synthesis and accelerates degradation, thus reducing chlorophyll content.

Meanwhile, previous studies had found that drought stress leads to increased accumulation of malondialdehyde (MDA) and peroxide dismutase (POD) in plants (Shao et al., 2006). MDA was a class of highly reactive lipid peroxides that cross-link and polymerize lipid nucleic acids, proteins, etc., and affected the components of cytoplasmic membranes, including chloroplast lamellae. POD can generate reactive oxygen species and trigger lipid membrane peroxidation under longer drought stress. Both substances can lead to changes in membrane structure and affect water metabolism by causing water loss in the chloroplasts, and thus the rate of chlorophyll synthesis was reduced. It has been suggested that drought stress can lead to reduced chlorophyll content in wheat, accelerated leaf senescence, and reduced green leaf area, resulting in reduced wheat yield (Verma et al., 2004). Also, previous studies had shown that the CC values of wheat flag leaves under drought stress tend to decrease and that wheat varieties with higher CC values under stress have higher dry matter quality and better drought resistance (Sun et al., 2019). These findings were consistent with the results of this article that the CC values of wheat flag leaves under drought stress showed a decreasing trend.

### Generality of CC Inversion Model

Current UAV multispectral with high spectral resolution and flexible mobility played an important role in crop high-throughput phenotyping studies. In this article, we use UAVs with multispectral cameras for ground image data acquisition and estimation of ground CC content. The reflectance extraction of the image data revealed that the spectral reflectance curves of different fertility stages studied in this article and the phenomenon of a green light wave peak at a wavelength of about 550 nm can be seen, and this result was more consistent with the results of the literature (Aasen et al., 2015). The positions of the green light peaks differed among the different fertility stages, with the wavelengths of the peaks appearing at the filling, flowering, and heading stages ranging from large to small. A red trough appeared between 630 and 670 nm, and the pattern of the red trough was consistent with that of the green peak. In the range of 466–830 nm, the reflectance of the multispectral data has high accuracy, and this result is more consistent with the results of the literature (Aasen et al., 2015).

A single vegetation index does not adequately reflect the crop growth, but too many vegetation indices as input parameters of the model will lead to an increase in the complexity of the model. Therefore, the optimal vegetation indices for different fertility periods were obtained by a random forest algorithm before model construction, and the vegetation indices for different treatments involved in model construction at different periods were determined by combining the correlation of CC and vegetation indices later. The vegetation indices involved in model construction under normal treatment at the heading stage were REOSAVI, VARIgreen, NDREI, NDVI, MSR, and SR, and vegetation indices under water limitation treatment were VARIgreen, OSAVI, NDREI, NDVI, and VARIred; the vegetation indices involved in model construction under normal treatment at the flowering stage were NDVI, GCI, VARIgreen, NDVI, and VARIred. GCI, VARIgreen, NDREI, MSR, and SR, and the vegetation indices under the water-limited treatment were REOSAVI, NDRGI, NDREI, NDVI, and MSR; the vegetation indices involved in the model construction under the normal treatment at flowering were VARIgreen, NDRGI, NDVI, EXG, and NGBDI, and the vegetation indices under the water-limited treatment were. The preferred vegetation indices under different water treatments in three different periods were different, but NDVI was not selected only in the water treatment during the filling period, while all other models were involved, which also indicates the prevalence of NDVI vegetation indices in crop model construction and the importance of NDVI vegetation indices. This is also consistent with many current types of research using NDVI vegetation indices in modeling studies.

In terms of CC prediction models, this article investigates CC prediction models by nine machine learning algorithmic models for three different fertility stages, namely, heading, flowering, and filling, under normal irrigation and water limitation treatments, respectively. The prediction models were found to be different for different water treatments at different fertility stages, but the model with the higher correlation between both predicted and true values under different treatments at different fertility stages was the SVM model, which embodied a strong fit and accuracy among all the models. The modeling of SVM under the normal treatment at the heading stage (*r* = 0.62, RMSE = 3.58, NRMSE = 17.3%) ranked second in correlation, and the modeling of SVM under the water limitation treatment (*r* = 0.63, RMSE = 3.47, NRMSE = 19.2%) ranked first in correlation; the modeling of SVM under the normal treatment at the flowering stage (*r* = 0.50, RMSE = 2.79, NRMSE = 17%), ranked second in correlation, modeling of SVM under water limitation treatment (*r* = 0.46, RMSE = 2.98, NRMSE = 18.6%), ranked second in correlation, and modeling of SVM under normal treatment at filling stage (*r* = 0.43, RMSE = 2.91, NRMSE = 21.6%), ranked first in correlation, and modeling of SVM under water-limited treatment (*r* = 0.51, RMSE = 3.57, NRMSE = 16.3%), ranked first in correlation. From the overall point of view, SVM showed the most advantage in the filling stage, this is related to the adaptability of the model under different water treatments at different fertility stages, that is, the prediction effect of different models applying different water treatments at different fertility stages is different. From the distribution of SPAD, the distribution of SPAD in the heading stage, flowering stage, and filling stage were significantly different, the distribution of the filling stage was more stable, and the time nodes of the population in the filling stage were more consistent with the fertility stage, and the prediction model of SVM had higher accuracy.

## Conclusion

The multispectral images acquired by the UAV were used to extract the reflectance of five spectra of different genotypes (Blue, Green, Red, Red_edge, and Nir) and calculate different vegetation indices, combined with the ground canopy data collected by the handheld CC instrument underwater and dry treatments at the heading, flowering, and filling stages. Then the relevant research analysis was carried out, and the analysis of the reflectance curve of the spectrum showed a phenomenon that the green wave peak appeared at around 550 nm, and the position of the red wave valley appeared once between 630 and 670 nm, obviously, and the occurrence law of the red wave trough is consistent with that of the green wave peak. It shows that the reflectance data obtained in this study are of high quality and have good accuracy. In the study of CC phenotype distribution, it can be found that the range of variation of this population under different water treatments at different fertility periods is large, the genetic variation of the population is rich and the CC content under normal irrigation is significantly higher than that of water-limited treatment. The vegetation indices under different water treatments at different fertility periods were selected by combining the preferred vegetation indices and the correlation evaluation of CC with vegetation indices. This study examined a series of machine learning algorithms, including Adaboost Regression, Bagging_Regressor, Gradient_Boosting_Regressor, K_Neighbor, Random Forest, SVM, Lasso, RidgeCV, and Ridge in the high-throughput phenotyping context. The results showed that the highest predicted correlation under normal irrigation at the heading stage was the RidgeCV model with correlation coefficient *r* = 0.63, which had RMSE = 3.28 and NRMSE = 16.2%, and the one with highest correlation under water limitation treatment was the SVM model with correlation coefficient *r* = 0.63, RMSE = 3.47 and NRMSE = 19.2%; under normal irrigation at the flowering stage, the highest correlation was from the SVM model, which had a correlation coefficient of *r* = 0.50, RMSE = 2.79, and NRMSE = 17%, and the model with the highest correlation was Bagging_Regressor under water restriction treatment, which had a correlation coefficient of *r* = 0.50, RMSE = 2.90, and NRMSE = 19.1%; and under normal irrigation at the filling stage, the highest correlation came from the SVM model, which had a correlation coefficient of *r* = 0.43, RMSE = 2.91, NRMSE = 21.6%, and also the SVM model has the highest correlation under water limitation treatment with coefficient of *r* = 0.51, RMSE = 3.57, NRMSE = 16.3%. The results of this study showed that the prediction model constructed using the SVM model under different water treatments at different fertility stages could better invert the chlorophyll content of winter wheat canopies with different growth differences.

Many machine learning and empirical models can be selected to correlate hyperspectral reflectance with CC; therefore. it was worth investigating which models worked better and whether the combination of individual regression techniques can provide better predictive performance. The study of CC model inversion by a large number of machine learning algorithms also provided a reference for machine learning in model prediction applications. The cumulative data obtained through field trials were still empirical models obtained through statistics, which have some limitations in the spatial and temporal domain. Altogether, our results provide insights into the capacity of UAV-based remote sensing for switchgrass high-throughput phenotyping in the field, which will be useful for breeding and cultivar development. Moreover, the UAV-based approaches proposed in this study, including the wheat's SPAD-phenotyping and predicting model, facilitated high-throughput, and precise phenotype mapping, which should have an impact on wheat breeding as well as practical use in the field. In the future, we will try to add environmental factors while improving the accuracy of UAV remote sensing images to reduce the limitation of environment on the model and give full play to the advantages of UAV high-throughput phenotype acquisition.

## Data Availability Statement

The original contributions presented in the study are included in the article/supplementary material, further inquiries can be directed to the corresponding author/s.

## Author Contributions

HG contributed to conceptualization, project administration, resources, supervision, writing the original draft, and writing–reviewing and editing the manuscript. WW contributed to formal analysis, writing the original draft, and writing–reviewing and editing the manuscript. YC, YR, and ZZ contributed to investigation, methodology, and data curation. All authors have read and agreed to the published version of the manuscript.

## Funding

The present study was funded by the Xinjiang Major Science and Technology Special Project (2021A02001-1).

## Conflict of Interest

The authors declare that the research was conducted in the absence of any commercial or financial relationships that could be construed as a potential conflict of interest.

## Publisher's Note

All claims expressed in this article are solely those of the authors and do not necessarily represent those of their affiliated organizations, or those of the publisher, the editors and the reviewers. Any product that may be evaluated in this article, or claim that may be made by its manufacturer, is not guaranteed or endorsed by the publisher.
